# Some Public Health Reports

**Published:** 1915-05-15

**Authors:** 


### A MIRROR OF FACTS.

### Some Public Health Reports.

Reports from medical officers of health can hardly claim to be attractive reading, yet they mirror facts which are of great economic and national importance. It is here may be found a representation of the conditions under which large numbers of our people live, the existing circumstances which favour disease on the one hand and promote health on the other, and the success or failure of legislative efforts intended to establish the well-being of the nation. The writers are generally men of experience and authority, and they aim without question at accuracy of statement. In some measure such reports must have a technical quality which hardly makes for popular perusal, and the same may be said of statistical information. These features cannot be avoided, but we could sometimes wish they were less prominent. Necessary for the purposes of the Local Government Board, they do not help the educational influence which such reports ought to serve. Possibly figures and technicalities could sometimes be relegated to an appendix, and the general body of the report would be phrased so as to promote popular instruction. There is some degree of danger that a section of the public may come to look upon the medical officer of health as an official person whose duty it is to detect shortcomings and find fault in mundane affairs, just as some persons come to regard in this light other officials charged with a less material mission. Such a result is to be avoided, for we want our health experts to be recognised as the friends of the people and not as their censors. They ought not to rest upon some lofty pinnacle from whence issue commands for the guidance of a public naturally unenlightened and slow of heart. Rather their ambition should be to invite the confidence which comes less to the official than to the practical helper and friend. The British public, we were once told, has no desire to be governed by professors, and the national taste in this respect is hardly likely to have been modified by recent events. John Bull, in short, may be led with a fair amount of ease, but to drive him is a difficult undertaking.

We have been prompted to make these remarks by the perusal of a number of recent reports collected from various parts of the country. In doing so it is far from our desire to assume the *rôle* of the censor. What we wish to secure rather is the full influence of much valuable work. The teaching, indeed, is excellent, but we are not sure that it is always presented in the fashion best suited to reach and stimulate the congregation. After all, it is public opinion which must be educated if improvements are to be secured. Neither national nor municipal legislation can be effective without the driving influence of this motive force.

It is fair to recognise the difficulties under which not a few medical officers of health labour both in their work generally and in the construction of their reports. The pressure of local vested interests is notorious, and he is a fortunate man who altogether escapes it. No one proposes to attack the principle or practice of local self-government as we know it in this country, but as some qualification of its benefits it certainly does afford an opportunity both to the nobodies and the busybodies. We have to try to suffer these inflictions as gladly as our temperaments permit; certainly for the couch of the medical officer of health they often provide something much more spiny than the proverbial crumpled rose-leaf. A victory over them rejoices all the friends of sound municipal government, and we notice one of these with much satisfaction. In a certain urban district, which shall be nameless, there exists, according to the local medical officer of health, "dirt, overcrowding, bad housing, ignorance, vicious habits, and the use of abortifacient drugs," and this unhappy catalogue was duly set forth in the officer's official report to the Local Government Board. Not altogether surprising was it to find that, when the report was presented to the district council, a councillor interested in the incriminated region arose in all his municipal majesty, denounced the paragraph in question in terms of indignant eloquence, and moved that it be deleted. But all this proved a vain show. The medical officer mildly explained that his report was an official communication to the Local Government Board, and that neither any individual councillor nor the Council in its corporate capacity had the power to dictate the terms in which it should be phrased. The responsibility rested upon himself, and not upon the Council, and he fully accepted it. Thereupon collapse of the indignant municipal individual, who, it is worthy of note, was deprived even of the sympathy of his colleagues. Obviously, if public health reports are only to speak the smooth things which are pleasant to the ear of those who cultivate local interests, their value and trustworthiness are at an end. Unless the medical officer is free to speak what he believes to be the truth, he had better be promptly brought to a compendious end.

A feature in several of the reports now before us is the announcement of diminished hostility on the part of the parents to the medical school inspection of their children. Evidently there is happening here what happened years ago in connection with the removal to hospital of children suffering from infectious diseases. Suspicion of the authorities and the noble claim of individual liberty were prominent at the outset, whereas nowadays the diagnosis is almost invariably followed by a prompt request for the hospital. Our public health colleagues do not receive much public recognition or public gratitude, but their work is providing the material necessary to the physical fitness and efficiency of the nation. Just now they are suffering, like the rest of us, from the invading and limiting influence of the war. This, however, will pass, and in the meantime it is necessary to sustain the influences which are essential to a healthy and vigorous national life.

